# Rapid fat quantification in *Torreya grandis* kernels using portable near-infrared spectroscopy: effects of shelling and kernel processing

**DOI:** 10.3389/fnut.2026.1830282

**Published:** 2026-04-21

**Authors:** Zidong Yang, Fansong Zeng, Yuqi Gu, Yu Huang, Huimin Fang, Muhammad Hassan, Lijian Yao, Chao Zhao

**Affiliations:** 1 College of Optical, Mechanical and Electrical Engineering, Zhejiang A&F University, Hangzhou, Zhejiang, China; 2 School of Agricultural Engineering, Jiangsu University, Zhenjiang, Jiangsu, China; 3 U.S.-Pakistan Center for Advanced Studies in Energy (USPCAS-E), National University of Sciences and Technology, Islamabad, Pakistan

**Keywords:** fat, PLSR, portable near-infrared spectroscopy, quantitative prediction, *T. grandis* kernels

## Abstract

Fat is a dominant nutritional constituent of nuts, significantly influencing their appearance, sensory properties, storage, and processing behavior. As a key quality attribute, rapid and accurate quantification of fat is essential. This study aimed to develop portable near-infrared spectroscopy (NIRS) models for the rapid determination of fat content in *Torreya grandis* (*T. grandis*) kernels. Three physical states of the kernels were evaluated: in-shell kernels, de-shelled kernels, and kernel granules. Spectra were acquired using a Smart Eye 1700 portable NIR spectrometer, while reference fat contents were determined using the traditional Soxhlet extraction method. Principal component analysis (PCA) was employed to explore spectral structures, and outliers were identified and removed using Mahalanobis distance combined with concentration residual analysis. Following outlier removal, the dataset was partitioned into calibration and prediction sets via the SPXY (sample set partitioning based on joint x-y distances) algorithm. Partial least squares regression (PLSR) models were then established utilizing various preprocessing strategies.The results demonstrated that the kernel granules provided the optimal prediction performance. The best-performing model, designated SG-SNV-PLSR-K, achieved a determination coefficient of the calibration set ( $R_{c}^{2}$ ) of 0.92 and a determination coefficient of the prediction set ( $R_{p}^{2}$ ) of 0.89. Compared to the optimal de-shelled kernel model (MSC-PLSR-DS), the granule-based model improved calibration and prediction accuracy by 3.99% and 12.65%, respectively. Furthermore, compared to the in-shell model (SNV-PLSR-IS), the accuracy improved by 16.32% and 20.36%, respectively.These findings indicate that portable NIRS, combined with chemometric analysis, offers an effective and high-throughput approach for fat screening in *T. grandis* kernels. The developed workflow not only facilitates rapid quality assessment for this specific species but also provides a transferable methodology for fat analysis in other nut varieties.

## 1 Introduction

With increasing public attention to food quality and safety, rapid and reliable evaluation of key nutritional components in agricultural products has become increasingly important (1, 2). In nut products, chemical composition is closely associated with quality attributes, market value, and consumer acceptance (3, 4). Fat content represents a critical quality metric for *T. grandis*, as it is a primary determinant of nutritional density and significantly dictates flavor profiles, visual appeal, and overall consumer satisfaction (5). A rapid, dependable fat assay is essential to facilitate quality grading and streamline the selection process for *T. grandis*. However, conventional analytical techniques, including Soxhlet extraction, acid or alkali hydrolysis, and the Babcock method, typically involve laborious sample preparation (such as dehydration and pulverization), extended chemical processing times, and high reagent toxicity (6). These destructive approaches generate substantial solvent waste and suffer from inherently low throughput, rendering them more appropriate for sporadic laboratory testing than for the high-speed inspection of large batches during commercial trade or industrial processing (7). There is consequently an urgent need for new, efficient fat quantification methods for *T. grandis*.

Near-infrared spectroscopy (NIRS) characterizes samples by capturing their unique optical signatures, specifically absorption, scattering, and reflectance, within the NIR spectrum (8–10). This technique is distinguished by its high speed, reagent-free nature, and minimal need for sample pretreatment (11, 12). Portable NIR spectrometers offer practical advantages for real-time, non-destructive detection due to their compactness, low cost, and fast operation (13, 14). By enabling in-situ, real-time diagnostics without rigorous sample preparation, they are suited to field detection, process monitoring and rapid commodity quality assessment (15, 16). For *T. grandis* in particular, this contactless and rapid quantification approach allows farmers and commercial processors to implement on-site, high-throughput fat content sorting during harvest and postharvest processing, enabling efficient quality grading, streamlined germplasm selection, and improved market value allocation. NIRS has been extensively integrated into agricultural and food science, specifically for the rapid profiling of fat-related parameters in various nuts (3). Recently, the synergy between NIRS and advanced chemometric algorithms, machine learning, and artificial intelligence has further broadened its utility across the agricultural, forestry, and food industries (17–21). These advancements encompass comprehensive food composition analysis, cultivar identification, quality standardization, and real-time process control (22–26). Empirical evidence has demonstrated that NIRS yields reliable fat content predictions across a spectrum of nuts and agricultural products. Notable benchmarks include studies on peanuts, almonds, walnuts, and corn, where diverse preprocessing techniques and modeling frameworks have been employed to enhance predictive accuracy (27–31). Among these biological matrices, peanut-based models have consistently exhibited superior robustness, highlighting the critical role of wavelength optimization and strategic variable selection (31). Empirical benchmarks include peanut models with $R_{p}^{2}$ values >0.97 through wavelength selection, and studies on almonds and walnuts which show that oil-rich matrices are well suited to NIR quantification, although shell interference and sample heterogeneity require well-chosen preprocessing for optimal precision (27, 29, 30). However, low-fat matrices like corn present a greater analytical challenge for NIR quantification due to their relatively attenuated lipid absorption signals (28). Notably, portable NIR devices achieve nearly the same performance as benchtop systems for major fatty acid prediction and composition identification (32). Collectively, these findings underscore NIRS as a highly promising modality for the accelerated quantification of fat in nut-derived products. However, research on *T. grandis* remains relatively limited, especially with respect to portable NIRS and the influence of different physical states, such as in-shell, de-shelled, and granulated samples on model performance. For the hard-shelled *T. grandis*, a commercially valuable nut with a thick, dense outer shell, this shell interference effect is hypothesized to be particularly pronounced, which raises critical questions about the extent of NIR light penetration through its hard shell and the resulting impact on spectral quality and model accuracy; this key knowledge gap directly motivated our study of these three different sample states.

In practice, *T. grandis* kernels may be presented in-shell kernels, de-shelled kernels, or processed kernel material. Such differences in physical state change light penetration and scattering behavior and therefore influence spectral quality and model performance. Specifically, the hard outer shell of *T. grandis* causes severe random light scattering and reflection, while the uneven surface of intact shelled kernels leads to milder yet notable light scattering and baseline drift; even kernel granules, despite improved homogeneity, exhibit minor particle size-induced scattering effects. These physical state dependent spectral artifacts obscure the characteristic absorption signals of fat in *T. grandis* kernels, which necessitates the employment of a diverse set of spectral preprocessing methods to eliminate non-chemical interference, correct spectral distortions, and enhance the signal-to-noise ratio of fat-related spectral features. Accordingly, this study collected portable NIR spectra of *T. grandis* under three states (in-shell kernels, de-shelled kernels, and kernel granules), used Soxhlet extraction fat values as references, removed outlier samples, and established PLSR models with various preprocessing methods. The aim of this study was to provide methodological guidance and data support for rapid, non-contact fat quantification in nuts.

## 2 Materials and methods

### 2.1 Samples and state definition

**Figure 1:**
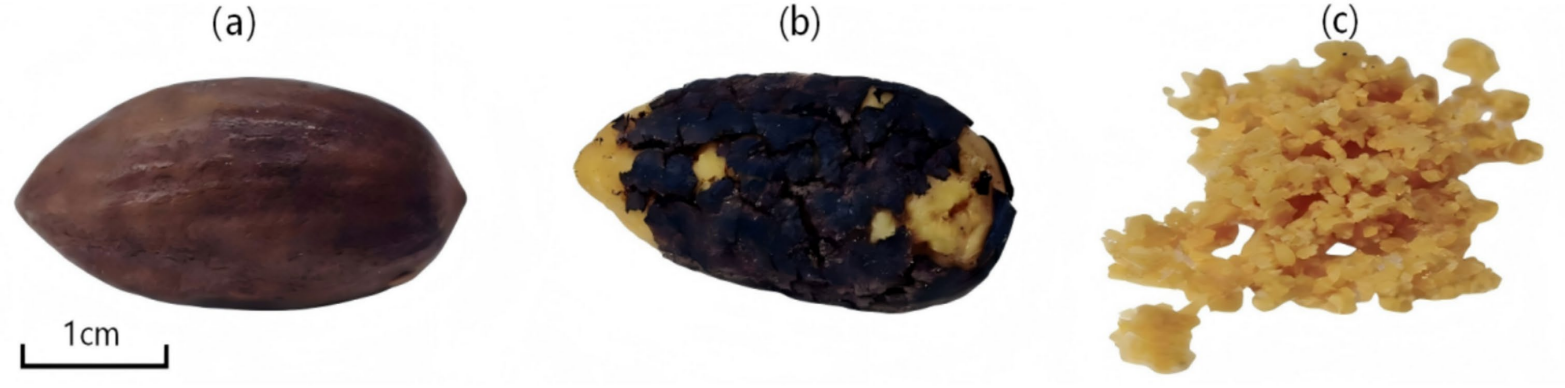
Three states of the *T. grandis* kernel. **(a)** *T. grandis* in-shell kernels; **(b)** *T. grandis* de-shelled kernels; **(c)** *T. grandis* kernel granules.

*T. grandis* kernels were collected from local farmers in Zhanao Village, Jidong Town, Zhuji City, Zhejiang Province, China. The harvest time was September 2021. Spectra were collected for three states: in-shell kernels, de-shelled kernels, and kernel granules (as shown in Figure 1). For fat content chemical value analysis, *T. grandis* kernels were shelled and the pseudotesta was removed to obtain kernels. The kernels were then ground into uniform fine granules with a particle size of approximately 40–60 mesh using a stainless steel high-speed universal grinder (RKT-010, Amoi Instrument Co., Ltd., Hebei, China) operating at a rotational speed of 2,800 r/min for 60 s to reduce variability caused by sample heterogeneity.

### 2.2 Portable NIR spectra acquisition

A Smart Eye 1700 portable NIR spectrometer (Huoyanjinjing Co., Ltd., Hangzhou, Zhejiang, China) was used for spectral acquisition. The spectrometer covered 1,000–1,650 nm with a 1 nm sampling interval and employed a dual integrated vacuum tungsten lamp (NVC Lighting, Huizhou, Guangdong, China) and a 128 element uncooled InGaAs diode array detector. Before scanning, the spectrometer was warmed up for 30 min. During measurements, laboratory temperature and relative humidity were maintained at 23 °C and 55%, respectively. A 100% Spectralon™ white reference panel was used for background correction. Measurement parameters were set to 50 averaged scans, 12.7 ms integration time, and 8 cm^−1^ resolution. Spectra were collected in the order of in-shell kernels, de-shelled kernels and kernel granules. Samples were positioned at the sampling window with vertical illumination. For each individual sample in each physical state, spectral measurements were conducted at three different spatial positions on the sample surface; the three replicate spectra obtained for each sample were then averaged to a single spectrum to minimize operational variability and improve the representativeness of spectral data. A total of 210 samples were scanned for each state. After scanning, kernel granules were stored refrigerated prior to chemical analysis.

### 2.3 Determination of fat content in *T. grandis* kernels

**Figure 2:**
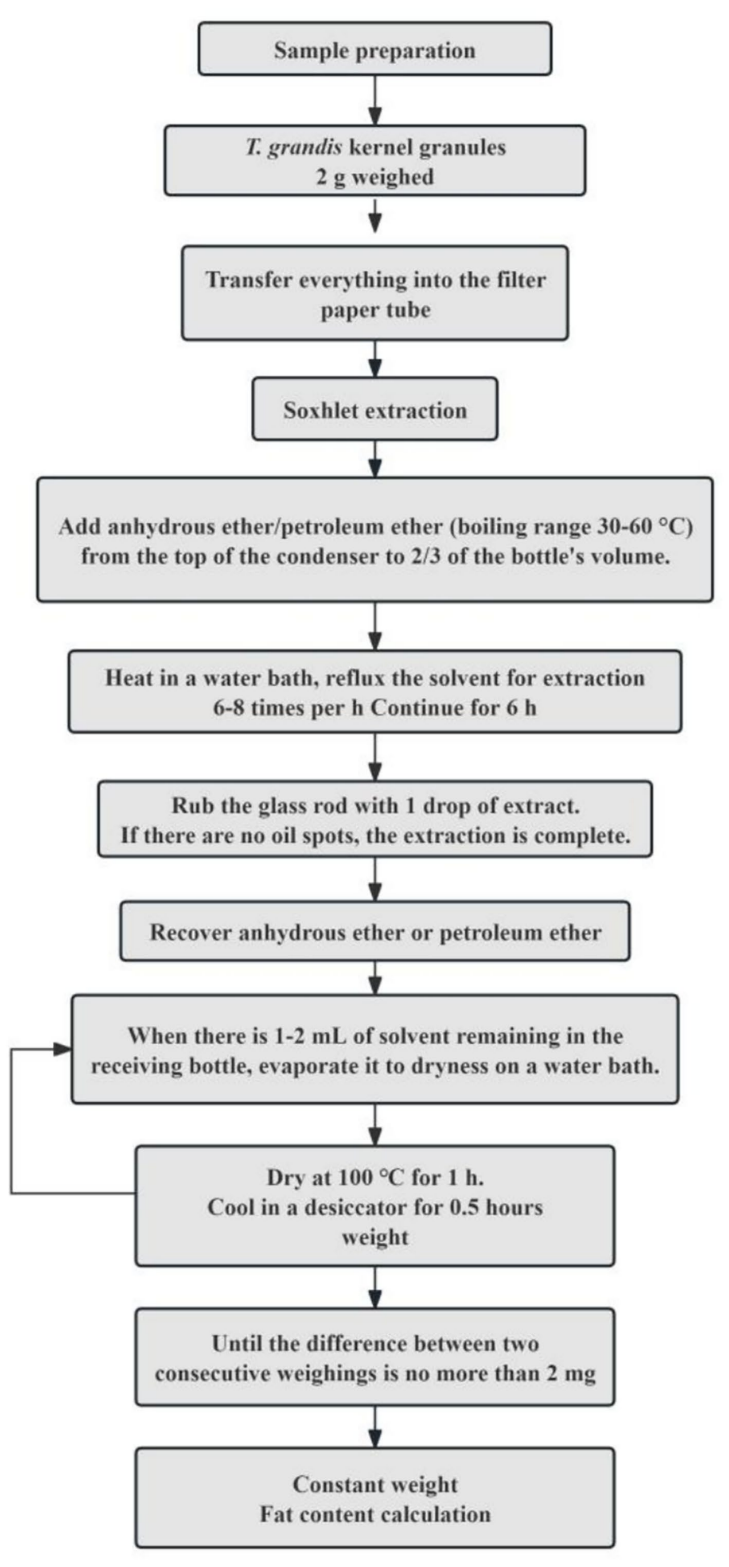
The schematic diagram of the Soxhlet extraction procedure.

Fat content was determined following “National Food Safety Standard: Determination of Fat in Foods” (GB 5009.6-2016), using the Soxhlet extraction method (see Figure 2 for the schematic diagram of the Soxhlet extraction procedure) (33). 2 g of *T. grandis* kernel granules was accurately weighed into a filter paper thimble. The thimble was placed into the extraction tube and connected to a receiving flask and a condenser. Anhydrous ether was added until the receiving flask was approximately two-thirds full. Extraction was performed for 6 h in a ~70 °C water bath with a siphon cycle of once every 3–5 min. After extraction, the thimble was removed and dried in a ventilated place for 1–2 h. The receiving flask was weighed repeatedly until constant mass.


$$F=\frac{\left(m_{1}-m_{0}\right)}{m}\times 100\%$$

where *F* is fat content (%); *m_1_* is the mass of the receiving flask plus extracted fat (g); *m_0_* is the mass of the empty receiving flask (g); and *m* is the sample mass (g).

### 2.4 PCA of NIR spectra

Raw spectra contain dense wavelength variables and thus high dimensionality, which can slow analysis and degrade modeling if redundancy and noise dominate (34). PCA is widely used to compress high-dimensional spectral datasets by projecting them onto orthogonal principal components that explain the largest variance, thereby retaining key spectral structure while reducing redundancy (35). Therefore, principal component analysis (PCA) was applied to the near-infrared spectra of *T. grandis* for dimensionality reduction.

### 2.5 Outlier sample removal

Outlier samples were grouped into two categories: spectral outlier samples and chemical outlier samples. Spectral outliers are typically associated with acquisition issues, whereas chemical outliers often originate from abnormalities during the chemical analysis procedure or data recording (36). In this work, PCA-Mahalanobis distance (PCA-MD) was used to identify and remove spectral outliers, and concentration residual analysis was used to remove chemical outliers. The first four principal components, explaining over 99% of cumulative variance, were used for Mahalanobis distance computation. The threshold for spectral outlier removal was set at the 95% confidence ellipse limit. For residuals, samples exceeding 2.5 times the standard deviation of chemical residuals were removed as outliers.

### 2.6 Dataset partitioning

After outliers removal, datasets were divided using the SPXY method. Because removed serial numbers and counts differed across the three states, and each state had distinct spectral behavior, each state dataset was partitioned separately into a calibration set (75%) and a prediction set (25%) with a ratio of 3:1 (3). As defined in the experimental design, X represented state-related spectral information and Y represented measured chemical fat values.

### 2.7 Model development and evaluation

Before model development, the spectra were preprocessed. The preprocessing methods used in this study included smoothing (Savitzky–Golay, SG), normalization (Normalize), standard normal variate (SNV), first derivative (1-Der), second derivative (2-Der), and combined preprocessing. An appropriate preprocessing strategy was selected to improve modeling accuracy (37). The accuracy of the quantitative prediction model for fat content in *T. grandis* was mainly evaluated using the determination coefficient ( $R_{c}^{2}$ ) and the root mean square error (RMSEC) of the calibration set. Here, $R_{c}^{2}$ described the fit of the model to the calibration data. The predictive ability of the model was evaluated using determination coefficient ( $R_{p}^{2}$ ) and the root mean square error (RMSEP) of the prediction set (16, 19). Higher $R_{c}^{2}$ and $R_{p}^{2}$ values, together with lower RMSEC and RMSEP values, indicated better regression performance.

## 3 Results and discussion

### 3.1 Determination results of fat content in *T. grandis* kernel

The fat content in *T. grandis* kernel determined by the Soxhlet extraction method is presented in Table 1. As shown in Table 1, the fat content in *T. grandis* kernel ranged from 40.85 to 75.43%, with a mean value of 54.63% and a standard deviation of 11.2. These results were close to previously reported fat contents for *T. grandis* kernel (54.62–61.47%) (38, 39). Notably, the range observed in this study covered and extended beyond the ranges reported in the literature, which improved the statistical representativeness of the sample set and supported more robust NIRS model calibration and development.

**Table 1:** Determination results of fat content in *T. grandis* kernels.

Component	Sample size	Maximum/%	Minimum/%	Average/%	Standard deviation
Fat	210	75.43	40.85	54.63	11.2

### 3.2 Portable NIRS characteristics of *T. grandis* kernels

**Figure 3:**
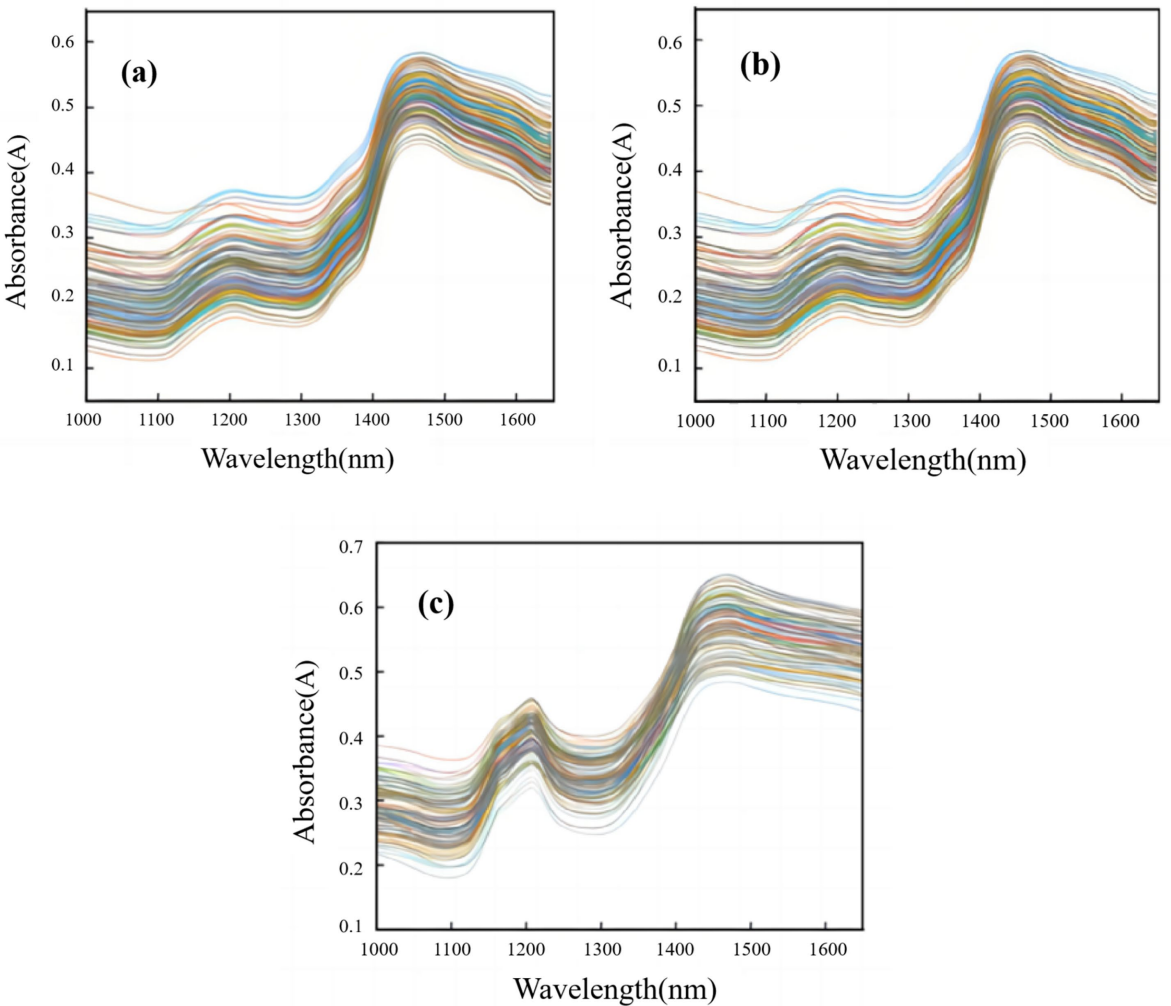
Portable NIR spectra of *T. grandis* kernels under different states: **(a)** *T. grandis* in-shell kernels, **(b)** *T. grandis* de-shelled kernels, and **(c)** *T. grandis* kernel granules.

Figure 3 shows the raw near-infrared spectra of *T. grandis* kernels under three states: in-shell kernels (a), de-shelled kernels (b), and kernel granules (c). As shown in the Figure, within the 1,000–1,650 nm range, two distinct absorption peaks were observed at approximately 1,200 nm and 1,450 nm. The peak near 1,200 nm was attributed to the stretching vibration of the C–O–C structure in ester groups and was considered as a key characteristic band of fat in *T. grandis* (31, 40). The peak near 1,450 nm was assigned to the stretching vibration of the amide (N–H) bond in fat and was generally associated with O–H and/or N–H related absorption bands, which may also reflect matrix effects associated with lipid-containing samples (27, 30). Overall, the three spectra exhibited similar trends; however, the absorbance of samples de-shelled kernels was higher than that of samples in-shell kernels, and the absorbance of kernel granules was higher than that of de-shelled kernels samples. This gradient of absorbance is closely related to the physical structure and surface properties of *T. grandis* kernels in different states. For in-shell kernels, the hard outer shell acted as a physical barrier, reducing the light penetration depth and the effective contact between NIR light and the kernel matrix. After shell removal, the physical barrier was eliminated, and NIR light could directly interact with the kernel. For kernel granules, the grinding process broke the intact kernel structure to form fine particles with a significantly larger specific surface area, maximizes the absorption of NIR light by fat and other components in the kernel, and thus results in the highest absorbance among the three states. Physical differences in surface and particle structure contribute significantly to the observed spectral differences. In summary, the absorption features of the *T. grandis* kernels spectra under three states were generally consistent with the wavelength ranges reported in the literature for fat related absorption, indicating that the measured spectra contained sufficient information for model development.

### 3.3 PCA results of near-infrared spectra of *T. grandis* kernels

**Figure 4:**
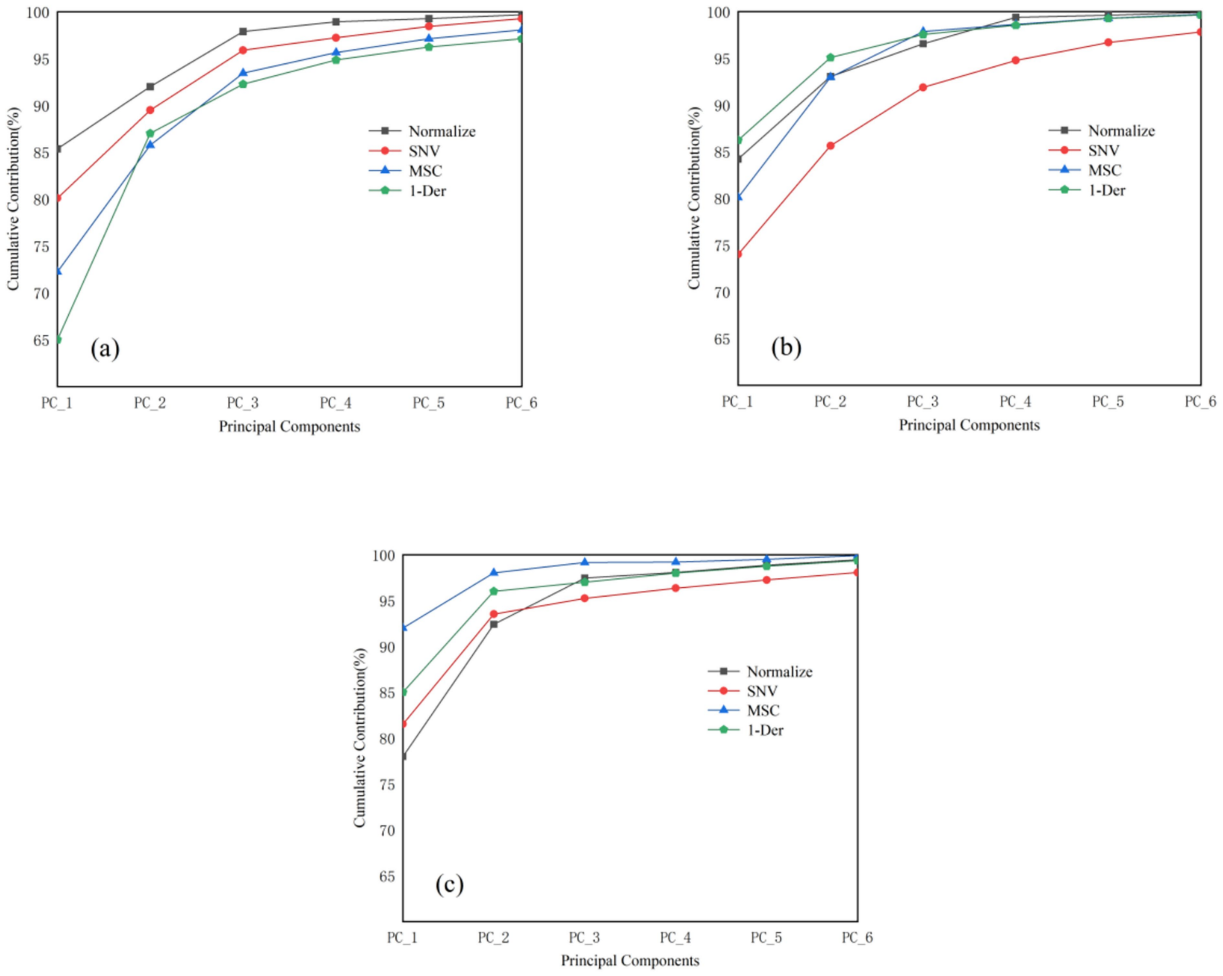
Cumulative contribution rate of the first six principal components of different preprocessing methods of portable NIR spectra: **(a)** *T. grandis* in-shell kernels, **(b)** *T. grandis* de-shelled kernels, and **(c)** *T. grandis* kernel granules.

The principal component analysis (PCA) results of the NIR spectra for *T. grandis* kernels under different states are shown in Figure 4: in-shell kernels (a), de-shelled kernels (b), and kernel granules (c). As shown in the Figure, for samples in-shell kernels, after Normalize preprocessing, the first principal component (PC1) explained 88.64% of the variance, and the cumulative contribution of the first four principal components reached 99.64%. Considering the clustering performance of the score plots, the scores of the first four PCs after Normalize preprocessing were selected for Mahalanobis distance calculation. For samples de-shelled kernels, after Normalize preprocessing, the cumulative contribution of the first four PCs exceeded 99%; therefore, the scores of the first four PCs after Normalize preprocessing were also used for Mahalanobis distance calculation. For kernel granules, after MSC preprocessing, PC1 explained 92.14% of the variance, and the cumulative contribution of the first four PCs reached 99.51%, which was the highest among the compared preprocessing methods. The first four PCs were selected for Mahalanobis distance calculation because their cumulative variance contribution rate exceeded 99%, a widely accepted threshold in chemometric analysis that ensures the retention of more than 99% of the original spectral information while effectively reducing data dimensionality, thus avoiding information loss and ensuring the reliability of outlier identification based on spectral characteristics. Accordingly, the scores of the first four PCs after MSC preprocessing were selected for Mahalanobis distance calculation.

### 3.4 Results of outliers removal

**Figure 5:**
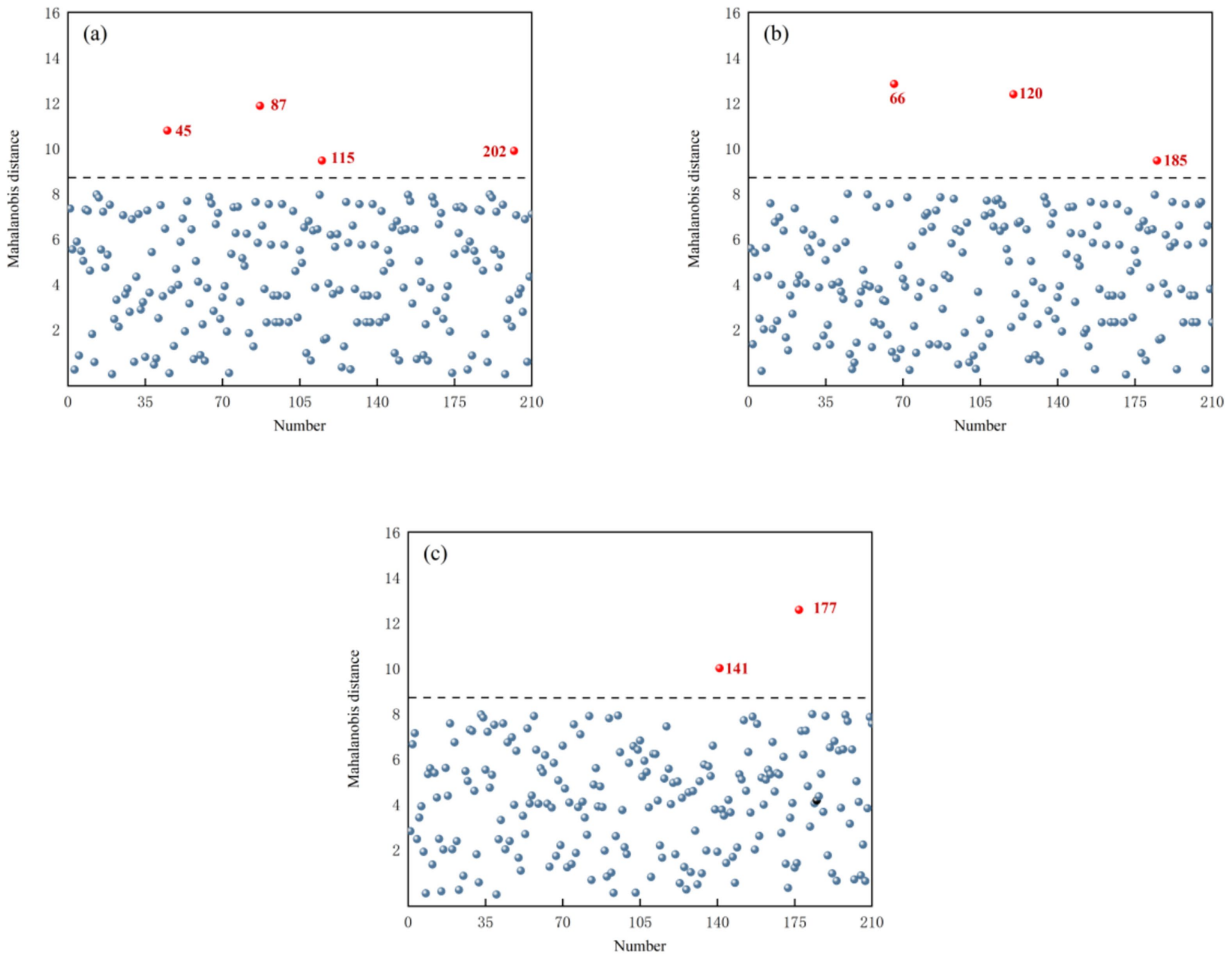
Map of Mahalanobis distance: **(a)** *T. grandis* in-shell kernels, **(b)** *T. grandis* de-shelled kernels, and **(c)** *T. grandis* kernel granules.

Based on the results in Section 3.3, Mahalanobis distances were calculated using the selected principal-component score matrices, and the resulting distributions are shown in Figure 5: in-shell kernels (a), de-shelled kernels (b), and kernel granules (c). Samples highlighted in red in the Figure were identified as outliers. As shown in Figure 5a, in the in-shell kernels dataset, Samples 45, 87, 115, and 202 exhibited excessively large Mahalanobis distances and were therefore identified as outliers (4 samples). As shown in Figure 5b, in the de-shelled kernels dataset, Samples 66, 120, and 185 showed excessively large Mahalanobis distances and were identified as outliers (3 samples). As shown in Figure 5c, in the kernel granules dataset, Samples 141 and 177 exhibited excessively large Mahalanobis distances and were identified as outliers (2 samples). The outlier removal results are summarized in Table 2.

**Table 2:** Elimination results of outlier samples of *T. grandis* kernels under different states.

Sample state	Method	Outlier number
*T. grandis* in-shell kernels	PCA-MD	45, 87, 115, 202
	Concentration residual	17, 66, 85, 156
*T. grandis* de-shelled kernels	PCA-MD	66, 120, 185
	Concentration residual	14, 105, 152, 182
*T. grandis* kernel granules	PCA-MD	141, 177
	Concentration residual	28, 85, 205

**Figure 6:**
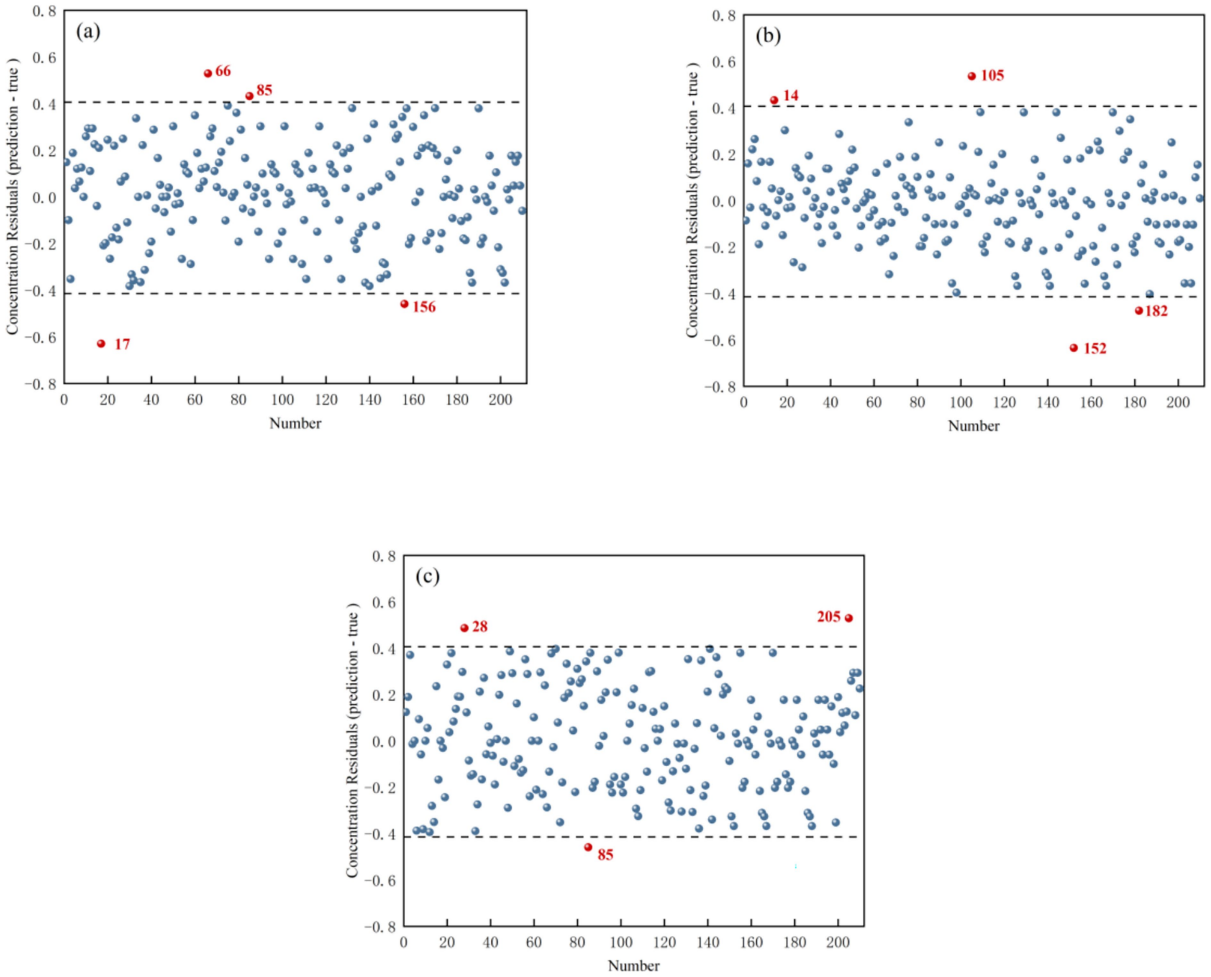
Spatial distribution of concentration residual. **(a)** *T. grandis* in-shell kernels, **(b)** *T. grandis* de-shelled kernels, and **(c)** *T. grandis* kernel granules.

Figure 6 shows the concentration residual distribution plots for *T. grandis* kernels in three states: in-shell kernels (a), de-shelled kernels (b), and kernel granules (c). Samples marked in red were identified as outliers. The removal results for abnormal chemical values were as follows: for samples in-shell kernels, Samples 17, 66, 85, and 156 were removed (4 samples); for samples de-shelled kernels, Samples 14, 105, 152, and 182 were removed (4 samples); and for kernel granules, Samples 28, 85, and 205 were removed (3 samples). The outlier removal results are summarized in Table 2.

### 3.5 Results of dataset partitioning for *T. grandis* kernels

The partitioning results of the NIR spectral datasets after outlier removal are shown in Table 3. Because the excluded samples differed among the three different states, and the spectra acquired in-shell kernels, de-shelled kernels, and kernel granules exhibited different spectral characteristics, the partitioning outcomes differed across the three different states. Overall, the fat content range of samples in the prediction set (41.73–73.23%) was narrower than that of the calibration set (40.85–75.43%), indicating that the dataset partitioning was appropriate. This helped to avoid model generalization issues caused by prediction samples falling outside the calibration range.

**Table 3:** Calibration set and prediction set for fat content in *T. grandis* kernels.

Sample state	Calibration set				Prediction set			
	Number	Range/%	Mean/%	SD/%	Number	Range/%	Mean/%	SD/%
*T. grandis* in-shell kernels	151	40.85–75.43	53.37	10.72	51	41.73–73.47	55.73	9.88
*T. grandis* de-shelled kernels	152	40.85–75.43	52.76	10.65	51	42.05–74.34	53.28	9.95
*T. grandis* kernel granules	153	40.85–75.43	54.38	10.81	52	41.80–73.23	55.67	10.03

### 3.6 Development and analysis of quantitative models for fat content in *T. grandis* kernels

The modeling results for fat content in *T. grandis* kernels under the three different states are presented in Table 4. As shown in Table 4, for *T. grandis* in-shell kernels, the PLSR model built on the raw spectra yielded $R_{c}^{2}$ and $R_{p}^{2}$ of 0.61 and 0.57, respectively, indicating relatively poor predictive performance. After 1-Der, 2-Der, and Baseline preprocessing, the model accuracy decreased to varying extents. This decrease might have occurred because these preprocessing methods reduced noise while also removing spectral information in fat-related wavelength regions. In contrast, models developed with other preprocessing methods showed improved prediction accuracy, indicating that appropriate preprocessing could still enhance model performance. Among them, SNV preprocessing produced the best performance. However, even the optimal model (SNV-PLSR-IS) showed the $R_{c}^{2}$ and $R_{p}^{2}$ below 0.80, which was insufficient for high-precision prediction of fat content in *T. grandis* kernels in-shell state.

**Table 4:** PLSR modeling results for fat content in *T. grandis* kernels under different preprocessing methods.

Sample state	Preprocessing method	Optimal number of latent variables	Calibration set		Prediction set			
			$R_{c}^{2}$	RMSEC	$R_{p}^{2}$	RMSEP	RPD	RER
*T. grandis* in-shell kernels	Original	6	0.61	1.23	0.57	1.29	1.52	24.60
	1-Der	6	0.61	1.24	0.57	1.30	1.51	24.42
	2-Der	7	0.60	1.26	0.56	1.32	1.48	24.05
	SG	6	0.62	1.20	0.57	1.27	1.54	24.99
	Normalize	6	0.63	1.18	0.60	1.22	1.61	26.02
	Baseline	5	0.56	1.34	0.54	1.39	1.41	22.83
	**SNV**	**8**	**0.79**	**0.93**	**0.74**	**1.00**	**1.96**	**31.74**
	MSC	7	0.66	1.12	0.63	1.18	1.66	26.90
	1-Der + SNV	7	0.62	1.19	0.64	1.16	1.69	27.36
	2-Der + SNV	8	0.71	1.04	0.69	1.08	1.81	29.39
	SG + SNV	8	0.69	1.07	0.67	1.11	1.77	28.59
*T. grandis* de-shelled kernels	Original	6	0.78	1.01	0.75	1.07	1.76	30.18
	1-Der	8	0.86	0.84	0.84	0.91	2.07	35.48
	2-Der	6	0.79	0.99	0.72	1.13	1.66	28.58
	SG	7	0.81	0.95	0.79	1.00	1.88	32.29
	Normalize	6	0.77	1.02	0.76	1.05	1.79	30.75
	Baseline	5	0.75	1.08	0.71	1.14	1.65	28.32
	SNV	7	0.80	0.97	0.78	1.02	1.84	31.66
	**MSC**	**8**	**0.89**	**0.76**	**0.79**	**0.98**	**1.92**	**32.95**
	1-Der + SNV	7	0.79	0.99	0.75	1.06	1.77	30.46
	2-Der + SNV	7	0.81	0.96	0.75	1.08	1.74	29.90
	SG + SNV	7	0.81	0.95	0.76	1.04	1.81	31.05
*T. grandis* kernel granules	Original	7	0.78	0.98	0.76	1.03	1.74	30.51
	1-Der	8	0.85	0.83	0.81	0.93	1.92	33.80
	2-Der	8	0.87	0.78	0.79	0.97	1.85	32.40
	SG	8	0.87	0.77	0.81	0.91	1.97	34.54
	Normalize	7	0.85	0.82	0.84	0.87	2.06	36.13
	Baseline	5	0.81	0.92	0.69	1.16	1.54	27.09
	SNV	8	0.86	0.80	0.83	0.89	2.01	35.31
	MSC	8	0.86	0.79	0.83	0.90	1.99	34.92
	1-Der + SNV	7	0.84	0.84	0.76	1.01	1.77	31.12
	2-Der + SNV	8	0.87	0.78	0.80	0.95	1.88	33.08
	**SG + SNV**	**9**	**0.92**	**0.66**	**0.89**	**0.80**	**2.24**	**39.29**

$R_{c}^{2}$ , the correlation coefficient of the calibration set; $R_{p}^{2}$ , the prediction correlation coefficient; RMSEC, the root mean square error of the calibration set; RMSEP, the root mean square error of prediction set; RPD, Relative Percent Difference; RER, Ratio of Error to Range; 1-Der, the first-order derivation; 2-Der, the second-order derivation; SG, Savitzky–Golay smoothing; SNV, Standard Normal Variate; MSC, Multiplicative Scatter Correction. The bolded row in this table represents the parameters of the optimal model.

For *T. grandis* de-shelled kernels, the PLSR model based on raw spectra achieved $R_{c}^{2}$ and $R_{p}^{2}$ of 0.78 and 0.75, respectively. Compared with the models in-shell kernels state, the predictive performance improved substantially. Except for baseline preprocessing, most preprocessing methods further improved model accuracy to different degrees. In particular, the PLSR model after MSC preprocessing (MSC-PLSR-DS) produced the highest $R_{c}^{2}$ (0.89), suggesting a small discrepancy between predicted and measured values and thus good predictive performance. The results indicated that the models developed by de-shelled kernels were more accurate and stable than those developed by in-shell kernels state. After shell removal, part of the NIR light could penetrate the pseudotesta and reach the kernel more directly, allowing kernel spectral information to be captured more effectively and reducing the loss of fat-related spectral features. This improvement in the deshelled state is consistent with previous NIR studies on nuts showing that removing the shell generally enhances model robustness and predictive accuracy. In almonds, shelled samples yielded clearly better cross-validation performance than in-shell samples for major fatty acids, with RPD values increasing from 1.73 to 2.40 for palmitic acid, from 1.73 to 2.16 for stearic acid, from 2.02 to 3.98 for oleic acid, and from 2.11 to 3.77 for linoleic acid, with the best results obtained for shelled almonds in dynamic mode (29). A similar tendency was observed for protein prediction in *T. grandis* kernels, where the optimal model improved $R_{c}^{2}$ from 0.69 in-shell state to 0.84 after shell removal (3). These findings support the interpretation that the shell introduces additional scattering and attenuation effects, thereby weakening the effective contribution of fat-related absorption features from the kernel.

For *T. grandis* kernel granules, the PLSR model built on the raw spectra yielded $R_{c}^{2}$ and $R_{p}^{2}$ of 0.78 and 0.76, respectively, and its performance was comparable to that of the de-shelled kernels model. Compared with the raw-spectra model, all preprocessing methods improved prediction performance to some extent. The optimal PLSR model for fat content prediction in *T. grandis* kernels was obtained with SG + SNV processing (SG-SNV-PLSR-K), giving $R_{c}^{2}$ and $R_{p}^{2}$ of 0.92 and 0.89, respectively. With both $R_{c}^{2}$ and $R_{p}^{2}$ exceeding 0.85, the quantitative prediction model showed good predictive capability for fat content in *T. grandis* kernel granules. After the kernels were ground into granules/powder, the more homogeneous material produced more complete spectral information under NIR illumination. Consequently, the model established in this state achieved the highest prediction accuracy and could essentially enable accurate prediction of fat content. The superior performance obtained for kernel granules also agrees with published evidence that sample homogenization markedly improves the quantitative capability of NIRS models. In the portable NIR study on protein in *T. grandis* kernels, the granulated state produced the best model among the three sample forms, with $R_{c}^{2}$ of 0.92, exceeding the corresponding values for both in-shell and without-shell samples (3). Related work on soybeans likewise showed that grinding and sample treatment substantially enhanced prediction performance, with the correlation coefficient increasing from 0.61 to 0.95 for crude protein and amino acid determination (41). While the investigation into chestnuts primarily contrasted whole fruits with their exposed internal matrices, rather than pulverized forms, it nonetheless established that calibration models derived from spectral data of opened specimens attained high-precision estimates for intrinsic components (42). Synthesizing these observations, it is evident that mitigating structural variations and enhancing specimen homogeneity facilitates the extraction of more authentic chemical signatures, consequently reinforcing the reliability of lipid quantification.

### 3.7 Comparison of NIRS models for fat content prediction

To contextualize the predictive efficacy of the optimized *T. Grandis* kernel model, its results were benchmarked against several prominent NIR studies focused on lipid quantification across diverse matrices (Table 5). While the inherent variability in sample types, lipid concentration ranges, spectral acquisition protocols, and validation frameworks necessitates a cautious interpretation, these investigations offer a substantive methodological baseline. Data synthesized in Table 5 reveal that NIR-based fat models for high-oil nuts typically yield performance ranging from moderate to exceptional (29–31, 38). Investigations into almonds and walnuts demonstrated satisfactory precision, affirming that oil-dense kernels are highly conducive to NIR-based quantification due to their pronounced C–H vibrational features (29). The recurrent application of derivative algorithms alongside SNV transformation in these studies underscores the necessity of such techniques in mitigating baseline instability and particle-size artifacts. Within the reviewed literature, the peanut-centric model exhibited remarkable stability, characterized by an $R_{p}^{2}$ of 0.97 (31). This high level of performance emphasizes the strategic importance of distilling informative wavelengths and streamlining regression architectures through redundancy elimination. It is noteworthy that the findings of Wang et al. For *T. grandis* kernels also yielded superior predictive power, with an $R_{p}^{2}$ of 0.98, outperforming the majority of nut-based studies summarized herein (38). Such high fidelity likely stems from the implementation of 2-Der filtering and CARS-driven variable screening, which collectively sharpened spectral resolution and isolated fat-sensitive spectral signatures. Conversely, the corn-based model produced lower predictive coefficients despite minimal absolute errors, illustrating the inherent analytical challenges of lipid detection in low-oil matrices where absorption signals are significantly attenuated. Overall, these comparisons indicate that NIR spectroscopy is particularly effective for high-fat nut matrices, while preprocessing strategy and wavelength selection remain decisive factors affecting model performance. Therefore, the present results further support the feasibility of NIR spectroscopy as a rapid tool for fat evaluation in *T. grandis* kernels.

**Table 5:** Comparison of representative NIRS models for fat content prediction in different matrices.

Number	Fat content/%	Sample	Preprocessing Method	Model	Calibration set		Prediction set			Reference
					RMSEC	$R_{c}^{2}$	RMSEP	$R_{p}^{2}$	RPD	
1	60	Almond	SNV + 1-Der	MPLS	0.80	0.94	1.09	0.88	2.61	(29)
2	58.12–74.25	“Persian” walnut	2-Der	MPLS	1.56	0.86	1.58	0.89	2.86	(30)
3	60.10–73.18	Walnut	SNV + 2-Der	PLSR	1.05	0.69	1.63	N/A	N/A	(27)
4	30.60–60.14	Peanut	Baseline	SPA-MLR	0.49	0.98	0.46	0.97	6.13	(31)
5	2.80–3.80	Corn	1Der	Deep learning	0.18	0.95	0.31	0.85	N/A	(28)
6	50.03–56.96	*T. grandis* kernels	2-Der + CARS	PLSR	N/A	N/A	0.03	0.98	7.91	(38)
7	40.85–75.43	*T. grandis* kernels	SG + SNV	PLSR	0.66	0.92	0.80	0.89	2.24	This study

$R_{c}^{2}$ , the correlation coefficient of the calibration set; $R_{p}^{2}$ , the prediction correlation coefficient; RMSEC, the root mean square error of the calibration set; RMSEP, the root mean square error of prediction set; RPD, Relative Percent Difference; MPLS, Modified partial least squares; SPA-MLR, Successive projections algorithm and multiple linear regression; 1-Der, the first-order derivation; 2-Der, the second-order derivation; SG, Savitzky–Golay smoothing; SNV, Standard Normal Variate; MSC, Multiplicative Scatter Correction; CARS, Competitive Adapative Reweighted Sampling; N/A, Not Applicable.

### 3.8 The predictive performance of the optimal quantitative model for fat content in *T. grandis* kernels

**Figure 7:**
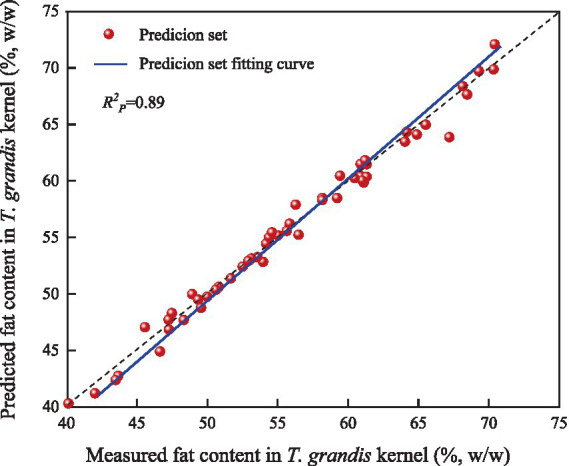
Correlations between the measured and predicted fat content in *T. grandis* kernel.

As described above, the near-infrared quantitative model established using spectra collected from the kernel granules (SG-SNV-PLSR-K) showed the best performance. The samples in the prediction set were therefore input into the model for verification. The correlations between the measured and predicted fat content in *T. grandis* kernels are shown in Figure 7. As shown in Figure 7, the X-axis represents the measured fat content in *T. grandis* kernels (%, w/w) and the Y-axis represents the predicted fat content in *T. grandis* kernels (%, w/w), with the solid line indicating the fitted regression line of the prediction set and scatter points representing the corresponding measured and predicted values of individual samples in prediction set. Most scatter points were distributed closely around the fitted regression line, with no obvious outliers, indicating that the predicted results were in good agreement with the measured values and confirming the predictive capability of the model. The fitted lines for the calibration set and prediction set were generally consistent. The $R_{p}^{2}$ was 0.89, indicating that the model exhibited good predictive performance. The model met the required prediction standard and was considered suitable for practical application. These results demonstrated that portable NIRS could be used for the rapid determination of fat content in *T. grandis* kernels.

## 4 Conclusion

In this study, *T. grandis* kernels were used as the research material, and portable NIRS combined with chemometric methods was employed to establish quantitative prediction models for fat content in three different states of *T. grandis* kernels: in-shell kernels, de-shelled kernels, and kernel granules. The results showed that the fat content in *T. grandis* kernels ranged from 40.85 to 75.43%. The NIRS data effectively provided the information required for quantitative modeling of fat content. For *T. grandis* in-shell kernels, the optimal quantitative prediction model for fat content was the PLSR model established after SNV preprocessing (SNV-PLSR-IS), with the determination coefficients of calibration and prediction sets of 0.79 and 0.74, respectively. For *T. grandis* de-shelled kernels, the optimal model was the PLSR model established after MSC preprocessing (MSC-PLSR-DS), with determination coefficients of calibration and prediction sets of 0.89 and 0.79, respectively. For *T. grandis* kernel granules, the optimal model was the PLSR model established after SG combined with SNV preprocessing (SG-SNV-PLSR-K), with determination coefficients of calibration and prediction sets of 0.92 and 0.89, respectively. Compared with the MSC-PLSR-DS model, the SG-SNV-PLSR-K model improved the accuracy of the calibration and prediction sets by 3.99 and 12.65%, respectively. Compared with the SNV-PLSR-IS model, the corresponding improvements were 16.32 and 20.36%, respectively. Under the optimal modeling conditions, the fat content prediction model for *T. grandis* kernels (SG-SNV-PLSR-K) showed sufficient predictive ability for rapid screening and preliminary quality evaluation.

## Data Availability

The original contributions presented in the study are included in the article/supplementary material, further inquiries can be directed to the corresponding author.
